# Modulation of Network Plasticity Opens Novel Therapeutic Possibilities in Cancer, Diabetes, and Neurodegeneration

**DOI:** 10.1002/advs.202522532

**Published:** 2026-01-31

**Authors:** Márk Kerestély, István Narozsny, Levente Szarka, Daniel V. Veres, Peter Csermely, Dávid Keresztes

**Affiliations:** ^1^ Department of Molecular Biology Semmelweis University Budapest Hungary; ^2^ Turbine Simulated Cell Technologies Budapest Hungary

**Keywords:** cellular learning, chromatin plasticity, endoplasmic reticulum stress, epigenetic memory, ferroptosis, intrinsically disordered proteins, molecular chaperones

## Abstract

Cellular plasticity is crucially important in cancer‐induced cell reprogramming, as well as in regeneration therapies in diabetes, Alzheimer's, and Parkinson's diseases. Protein–protein interaction, signaling, and gene regulatory networks are increasingly used to describe plasticity‐induced cellular adaptation in disease progression. This review delineates how network analysis of cell plasticity leads to novel therapy options against 1) cancer progression; 2) epithelial–mesenchymal transition‐induced metastases; 3) cancer stem cells, and 4) pre‐existent drug‐resistant cells. Network plasticity‐designed sequential and differentiation therapies are also outlined. 55 plasticity‐related cancer drug targets are listed, where 20 have already approved drugs, 9 have investigational drugs, and 26 are drug target candidates. The recent expansion of plastic network‐driven pancreatic beta cell and neuron regeneration therapies is described in diabetes, as well as in Alzheimer's and Parkinson's diseases, respectively. Finally, six major network‐related research gaps and promising future research areas are outlined, including the discovery of plasticity‐related cancer signaling pathways and cross‐talks, cancer resensitization therapies, and the use of recently available proteome‐wide network data and models to find novel cancer cell differentiation cocktails, drug targets, proper timing, and biomarkers of sequential therapies, as well as to perform in silico drug combination screens and in silico clinical trials.

## Graphical abstract

Plasticity changes of molecular networks form a cellular learning process. Signaling network plasticity promotes cancer, metastasis, and drug resistance development. 55 plasticity‐related cancer drug targets are listed (20 having already approved drugs, 9 investigational drugs, and 26 being drug target candidates). Plastic network–driven pancreatic beta cell and neuron regeneration therapies revolutionized diabetes, Alzheimer's, and Parkinson's disease treatments.

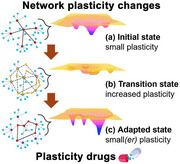

## 1 Introduction

**FIGURE 1:**
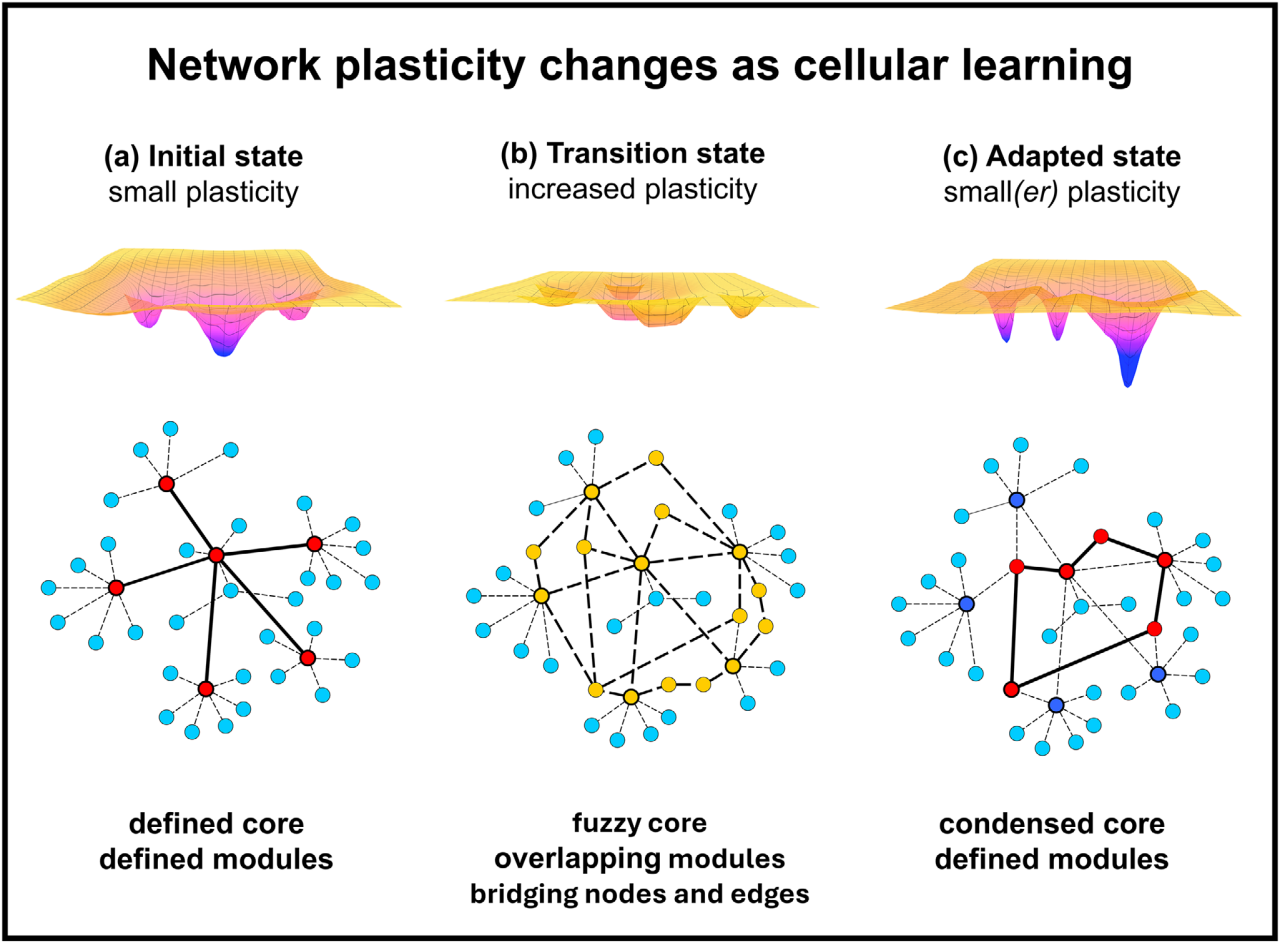
Network plasticity changes as a cellular learning process. Upper images: illustrative stability landscapes, with local stability minima acting as attractors. Lower images: corresponding illustrative network structures. We note that the figure shows a differentiation‐type adaptation, where the plasticity of the adapted state is lower than that of the initial state. In dedifferentiation, the situation is just the inverse.

Cellular plasticity refers to the ability of the same cell to produce different phenotypes in response to environmental changes. Plasticity plays a key role in embryonic development, tissue remodeling (e.g., wound healing), and disease‐induced cellular reprogramming, such as in cancer, diabetes, and neurodegeneration [1, 2, 3, 4, 5, 6, 7, 8]. As a sign of its increasing importance in therapeutic options, phenotypic plasticity has been recently incorporated into the hallmarks of cancer [4]. A balance between stability and plasticity is often regarded as a stabilizer of cellular states. However, increased plasticity of complex systems generally characterizes their adaptation to changes in their environment [1, 2, 3, 4, 5, 6, 7, 8, 9]. A system with an established, stable (relatively rigid) structure is crucially important for the precise execution of “business as usual” tasks of its everyday life [10, 11]. However, when adaptation is needed to a new situation, the system (or at least a dedicated part of it) must become plastic [2, 10, 11]. Therefore, an initial increase of system plasticity (followed by a later decrease) accompanies most cellular adaptive processes, which can be perceived as a cellular learning process (Figure 1) [11, 12, 13, 14, 15, 16, 17, 18]. Cancer development is one of the processes exhibiting a biphasic plasticity increase and decrease during cellular adaptation. First, rapid environmental changes, hypoxia, and immune attacks (all of which are often observed in cancer cells) induce considerable stress, leading to increased network plasticity in developing cancer cells [3, 4]. However, building a supportive niche by metastasized cancer cells (or by cancer stem cells) reduces these burdens and leads to decreased network plasticity [3]. Similarly, biphasic adaptation to high glucose concentrations may be observed in diabetes (see Section 4) [3].

A profound example of pioneering cell plasticity studies led to the 2012 Nobel Prize in Physiology or Medicine, which was awarded jointly to John B. Gurdon and Shinya Yamanaka “for the discovery that mature cells can be reprogrammed to become pluripotent” [19, 20]. The landmark discovery of the tetrad of OCT4, SOX2, KLF4, and c‐MYC transcription factors opened new ways of regenerative medicine, such as pancreatic beta cell regeneration in diabetes, or neuron regeneration in Alzheimer's and Parkinson's diseases [20, 21, 22, 23, 24].

Network representations provide a rich yet well‐organized data structure for complex systems from macromolecules to societies. Cellular functions are often described by protein–protein interaction networks, by signaling networks, and by gene regulatory networks [25, 26]. Complex systems having a plastic network structure can reside in multiple states having relatively similar stability and are divided by small energy barriers. In other words, network plasticity enables a “smooth” (flat) energy landscape, where several transitions may occur between individual system states, such as between protein conformations, cellular phenotypes, healthy and diseased cells, etc. Network plasticity can be assessed by defining a measure to characterize the order in the network, such as network entropy [27, 28, 29]. A recent study showed that attractors (i.e., grossly populated network states) are often protected by deep valleys. However, ridges between attractors often became flattened, facilitating network plasticity when multiple attractors co‐exist [30]. Plastic network structures are characterized by decreased (local or global) connection density, by a large, fuzzy network core (having a blurred transition to network periphery), by overlapping, fuzzy network modules, and by network nodes (also called “creative” nodes) connecting otherwise distant network nodes [26, 28, 29, 30, 31, 32].

This review summarizes the rapidly increasing recent knowledge on plastic networks and answers the following questions. 1) How do plasticity changes of protein structure networks and molecular networks of the cell contribute to cellular adaptation? 2) How does the plasticity of molecular networks promote cancer development? 3) How does the plasticity of the epithelial–mesenchymal transition help the formation of cancer metastases and cancer drug resistance? 4) Why does the extreme plasticity of cancer stem cells make them a special burden in anticancer therapies? 5) What is the role of network plasticity in the development of cancer drug resistance? 6) Why has network plasticity become an important, recently expanding area of cancer drug design? 7) What are the benefits of network plasticity in the treatment of diabetes? 8) How does network plasticity help regeneration therapies in Alzheimer's and Parkinson's diseases? In conclusion, the research gaps and perspectives of “plasticity drugs” (i.e., drugs developed by using the rapidly increasing knowledge on cell and network plasticity changes) will be delineated.

## 2 Network Plasticity as a Cellular Learning Process

Cellular adaptation involves cellular learning and cellular forgetting steps [12, 13, 14, 15, 16, 17, 18, 33]. Both processes involve changes in cellular plasticity [13, 17, 28, 29, 33]. In this section, first, the plasticity of protein structures and their network representations will be reviewed. This will be extended by an overview of plasticity changes in molecular networks of the cell, such as protein–protein interaction networks, signaling networks, and gene regulatory networks. Already, the plasticity of protein structures can provide important information on various diseases, such as cancer or neurodegeneration. This is grossly surpassed by the frequent use of molecular networks characterizing the development of cancer, cancer drug resistance, as well as Alzheimer's and Parkinson's diseases.

### 2.1 Plasticity of Protein Structure Networks

Protein structures can be perceived as networks of amino acids, featuring a tightly packed core and a plastic periphery [26]. Ligand binding to proteins extends the rigid core, unveiling a surprisingly long‐range contribution of four amino acid residue layers [34]. However, the entire protein molecule behaves as a single unit. Analysis of NMR studies revealed that in the native conformational state of a protein, the protein is in a critical state, where the motion of each amino acid residue is “felt” by every other residue. This means that despite being at a stable point in the energy landscape, the protein molecule still preserves its dynamic plasticity [17, 35]. Proteins integrate incoming information by maintaining a balance between their rigid core and plastic periphery, displaying a molecular “intelligence” [17]. Importantly, adaptive changes in the highly cooperative amino acid ensemble of a protein structure occur preferentially at mutations located at the protein periphery, distant from the ligand binding site. Conversely, the amino acid “bridge” connecting the distant mutation site and the ligand binding site is evolving cooperatively, preserving its bridging function [36].

Plasticity of proteins is increased by 1) intrinsically disordered protein regions, by 2) integrative plasticity of multiple phosphorylation events, and by 3) increased intracellular water content (decreased molecular crowding in the cell) [37]. Molecular chaperones, a class of proteins that help the folding of other proteins and RNAs, often have an intrinsically disordered region, which provides the extra plasticity to maintain their chaperone function [38]. Importantly, both major classes of molecular chaperones (the HSP60 and the HSP70 classes) display an ATP‐dependent “chaperone‐cycle,” where they first expand the unfolded protein (grossly reducing its plasticity) and then release it (giving a sudden burst of plasticity). These expansion/release cycles may be repeated several times [29, 39, 40, 41].

Intrinsically disordered proteins (IDPs) play an important role in cancer‐specific cellular events, serving as a source of increased cellular noise in cancer [28]. Several IDPs (such as JAG1, NOTCH, HIF1A, AKT, GSK3B, and TCF/LEF) play a key role in the epithelial–mesenchymal transition, a process leading to cancer metastasis and drug resistance [13]. 12 out of the 55 drug targets and potential drug targets (22%) listed in Section 3.5. as modulators of cancer cell plasticity are IDPs with a higher disorder content than 20% [42]. The IDP drug targets are master transcription factors of cancer plasticity (FOS, ASCL1, MYC, NRF2, FOXM1), cancer plasticity signaling pathway members (YAP1, GSK3), cancer cell differentiation proteins (PPAR‐gamma), regulators of partial epithelial–mesenchymal transition (NRG1), and modulators of chromatin plasticity (BDR3, BRDT, HDAC1). PTEN, a tumor suppressor, was identified as an IDP. Both the primary and secondary interactome layers of PTEN are enriched in IDPs, most of which are also cancer‐related [43]. Amyloid‐beta and alpha‐synuclein—the major players of Alzheimer's and Parkinson's disease, respectively—are IDPs [44, 45, 46]. 27 proteins potentially relevant to the amyloid cascade signaling pathway are IDPs [46]. These findings highlight the importance of molecular‐level plasticity in the development of plastic cellular phenotypes in cancer and neurodegeneration.

### 2.2 Plasticity of Protein–Protein Interaction, Signaling, and Gene Regulatory Networks

Molecular networks of the cell (i.e., protein–protein interaction networks, signaling networks, and gene regulatory networks) have much fewer physical constraints than protein structures (since, on the contrary to the protein backbone, they do not contain a continuous connection of covalent bonds). Therefore, network plasticity is displayed more at the cellular level than in individual proteins. The plasticity of cellular networks leads to plastic cellular phenotypes [8, 29, 47, 48]. However, plasticity at the molecular level (in the form of the already mentioned chaperones and IDPs, but also in the form of noncoding RNAs) greatly contributes to plasticity at the molecular network level [37].

Developmental plasticity leads to alternative phenotypes in response to environmental changes. Vandermeulen and Cullen [5] demonstrated that molecular networks display decentralized control, where different pathways become dominant under varying environmental conditions. Gene by Environment Interactions (GEI) network analysis of different environmental conditions may uncover a major regulatory role of a previously underappreciated pathway [5]. Importantly, different environments induce a grossly differing set of pathway cross‐talks [5, 8]. These observations underlie the importance of dynamic network nodes connecting distant network regions (like the “creative nodes” mentioned in the introduction) [31, 32]. As an example of developmental plasticity changes (similarly to the chaperone‐cycle we described in Section 2.1), differentiating progenitor cells first display a plasticity increase (leaving the relatively stable state of progenitor status), and only towards the end of differentiation (approaching the differentiated state), they become less plastic than the initial progenitor state [27].

Signaling networks need to be especially sensitive to environmental stimuli; therefore, they display increased plasticity in response to a changing environment. A mass spectrometry‐based phosphoproteomics study showed that breast cancer signaling networks treated with PI3K or mTORC protein kinase inhibitors became heterogeneously resistant to the same inhibitor [47]. These findings revealed that even under identical and carefully controlled experimental conditions, the evolution of distinct protein kinase network states took place. This indicated a high level of network plasticity. Context‐dependent kinase interactome changes also characterized the development of cancer plasticity in 18 diverse cancer cell lines [48]. Similarly, plasticity of the epithelial–mesenchymal transition (which leads to cancer metastases and drug resistance) revealed multi‐stable signaling and kinase interactome networks, where positive feedback loops and key signaling proteins (like AAK1, the adapter‐associated kinase 1) became especially important [48, 49]. Peripheral proteins of gene regulatory networks play a prominent role in the development of Alzheimer's and Parkinson's disease and are often connected to network hubs [50, 51]. This is a typical network configuration increasing network plasticity [10]. These studies demonstrate that molecular networks of the cell exhibit highly versatile forms of network plasticity, which opens the way to use this network feature in the development of new therapeutic options. In Sections 3 through 5, we summarize these new drug development areas in cancer, diabetes, and neurodegeneration.

## 3 Modulation of Network Plasticity in Cancer

The development of cancer can be viewed as a learning process, where malignant transformation and survival induce a plastic phenotype [13, 14, 15, 28, 52]. Phenotypic plasticity was included among the hallmarks of cancer in 2022 [4]. Differentiated cells lock their developmental plasticity. This process is accompanied by the locking of continuing cell proliferation, one of the key characteristics of cancer cells. Cancer‐acquired cellular plasticity may include dedifferentiation towards progenitor‐like cell states (which may lead to the development of cancer stem cells). Conversely, cancer cells may develop by disrupting the cell differentiation process and remaining in a partially differentiated, progenitor‐like, plastic stage [1, 2, 29]. Cancer cells themselves have interconverting subpopulations, which become remodeled upon drug treatment, leading to drug resistance [3, 53]. Cancer cell subpopulations get stabilized and reside in a cancer attractor [54]. As a clear sign of extreme plasticity, a subpopulation of cancer cells repopulated the whole original basin of attraction within days [55]. Increased cell heterogeneity and rearrangements of the signaling network are key features of plastic cancer cells [28, 56]. In this section, we first summarize our understanding of the increased plasticity of molecular networks in cancer and the network‐related therapeutic possibilities. Next, we describe the plasticity of the epithelial‐mesenchymal transition and its consequences in cancer therapy. We delineate the therapeutic options involving the extremely plastic state of cancer stem cells and the plasticity of drug resistance development. Finally, we encapsulate the plasticity‐related therapeutic options.

### 3.1 Plasticity of Molecular Networks in Cancer

Protein–protein interaction, signaling, and transcription factor networks in cancer cells can be characterized by increased plasticity, i.e., the ability to undergo rapid network reconfigurations in response to external stimuli [28]. Plastic networks of cancer cells become enriched in intermodular edges connecting different protein mega‐complexes and signaling pathways [56, 57, 58]. Transcription factor networks also increase their plasticity in cancer development [59]. Cancer cell plasticity is regulated by master transcription factors, such as HOXA5, SMAD4, and MITF [4]. Cancer‐related plasticity‐inducing signaling pathways, such as the NOTCH pathway, and pathways related to master transcription factors, offer promising therapeutic options for slowing down or eradicating cancer cells with increased plasticity [60, 61, 62, 63, 64, 65, 66].

#### 3.1.1 Plasticity of Cancer Interactomes and Signaling Networks

Altered interactome modularity was shown as a predictive measure in breast cancer patients [67]. As a sign of network modularity‐related plasticity increase, hubs with cancer‐specific mutations are located in the center of protein–protein interaction network modules, forming bridges between them [56], and tend to form a “rich‐club,” i.e., become connected with each other [25]. As examples of these phenomena, cancer interactomes become enriched by intermodular hubs, such as IRS1 or BRAF [25, 67].

Signaling networks of cancer cells contain more cross‐talks between signaling pathways than those of healthy cells [58]. Cross‐talks correspond to plasticity, increasing intermodular connections. For example, cancer‐specific cross‐talks bridge the RAS‐ERK and PI3K‐mTORC pathways, which makes drug combinations that inhibit both pathways an important therapeutic modality [68]. Cross‐talks may offer novel drug combination options, e.g., in cancer [69]. As another indication of increased signaling network plasticity, a higher entropy of signaling network degrees was found to correlate with poorer survival outcomes in prostate cancer patients [70].

An increase in signaling network plasticity needs the formation of new, “noncanonical” network connections in cancer cells. In a receptor tyrosine kinase network, the “noncanonical” ERBB1‐IRS1 connection became stronger in breast cancer cell lines as a summative result of numerous changes in the receptor tyrosine kinase network [32]. Another example is the inhibitory cross‐talk between the PI3K‐AKT and RAS‐ERK pathways, which leads to melanoma formation. First, benign changes induce RAS‐ERK signaling to such constitutively high levels that cell cycle arrest or senescence occurs. Secondary mutations activating the PI3K‐AKT pathway dampen RAS‐ERK signaling via AKT‐^V600E^BRAF phosphorylation to levels that cooperate with AKT to induce malignant transformation [68]. Aurora kinase A (AURKA) drives a non‐canonical crosstalk between YAP1 and TAZ, sustaining primary resistance to anti‐EGFR therapy in colon cancer [71]. The formation of “noncanonical” network connections is often extended by the development of “creative nodes” (i.e., fluctuating nodes giving transient connections between otherwise distant network modules)—a general characteristic of all cellular learning processes [26, 31]. Good examples of these creative nodes are intermodular, dynamic data hubs [26] and “critical nodes” (such as the IRS, PI3K, and AKT families) having several isoforms involved in divergent signaling, being highly regulated and forming cross‐talks [72]. Molecular chaperones (such as Hsc70, Hsp90, and Grp94) emerge as highly efficient date hubs [31] involved in the progression of cancer (and neurodegenerative diseases). Intrinsically disordered regions of these chaperones become phosphorylated, which turns them into an “epichaperone,” i.e., a protein that provides a multimeric scaffold rewiring protein–protein interaction networks [73]. Importantly, new plasticity‐increasing network connections may also originate from the tumor microenvironment in forms of direct cancer cell–stroma interactions or via exosomes [74, 75].

#### 3.1.2 Plasticity of Transcription Factor Networks in Cancer

Transcription factors are key regulators of the plasticity increase in a malignant phenotype. The work of Pillai and co‐workers [59, 76] identified a 17‐node transcription factor network that acts as a master regulator of melanoma development. Dynamic simulations of the network revealed that the network becomes separated into two modules centered around MITF (microphthalmia‐associated transcription factor; surrounded by 2 key other transcription factors, including FOS) and around JUN (surrounded by 7 transcription factors, including SMAD3 and KLF4). This network settled into 4 attractors (stable transcription factor expression patterns) corresponding to two proliferative and two invasive phenotypes. Simulations recapitulated major phenotypes observed in melanoma and explained the dedifferentiation trajectory after BRAF inhibition [59] as well as predicted an effective combination drug treatment strategy involving MITF and SMAD3 inhibition during BRAF inhibitor treatment. Simulations helped to quantify intra‐tumor and inter‐tumor heterogeneities and gave a possible explanation for multiple trajectories towards drug resistance [76]. The key role of JUN and FOS in the development of diverse plasticity patterns in melanoma cells has also been reported by Comandante‐Lou et al. [77]. These studies have shown that the increase in plasticity of transcription factor networks grossly contributes to the development of plasticity in cancer cells. A similar bimodal transcription factor network (centered around ASCL1 and NEUROD1) has been reported in connection with the phenotypic plasticity of small cell lung cancer. The two modules of this network inhibit each other. However, intra‐modular connections activate the modules. Thus, this transcription factor system forms a toggle switch [78]. In hepatocellular carcinoma, FOXM1 and CEBPB form a master decision‐making toggle switch, where the inhibition of FOXM1 restores tumor developmental homogeneity and re‐exposes tumor cells to immune surveillance [79]. As a recent study showed, toggle switch structures can be generalized to two mutually inhibiting teams of nodes [80].

HOXA5, SMAD4, and MITF are master regulator transcription factors modulating network plasticity [4]. HOXA5 is involved in the anticancer action of retinoid acid and DNA‐methyltransferase inhibitors in breast, colorectal, bladder, and prostate cancers [64]. Imatinib, a commonly used therapy in leukemia, blocks tyrosine phosphorylation of SMAD4 and restores TGF‐β growth‐suppressive signaling [65]. MITF is a quinacrine‐inhibited molecular driver of MAPK‐inhibitor‐resistant melanomas [63]. Recently, the circadian regulator/nuclear receptor REV‐ERB‐alpha was shown to be a master regulator of androgen receptor inhibitor‐induced plasticity increase in adenocarcinoma to prostate cancer transition. Rev‐ERB‐alpha switched its targets from kinase signaling and metabolic programs to increasing cell plasticity and inducing EMT and stem cell formation [81].

The transcription factor INSM1 plays a key role in glioblastoma differentiation towards neuronal cells. Inhibition of INSM1 inhibits the tumorigenicity of glioblastoma formation [82]. Another recent study suggested the transcription factor DEBPD, as well as TNFR1 and ITGB1, as mesenchymal hotspots and actionable nodes warranting therapeutic exploration in glioblastoma treatment [83]. Additionally, a subnetwork of FN1, CD44, and YAP1 was upregulated by mir199a‐3p associated with the mesenchymal phenotype of neuroblastoma [84].

SNAIL, ZEB, and TWIST are additional master transcription factors inducing cell plasticity and cancer progression. Their signaling networks drive cell dedifferentiation involving chromatin structure remodeling. These transcription factors are crucial components promoting the invasive potential and stemness of cancer cells [2]. ZEB2 has been shown to act as a key mediator of the transcriptional network related to cancer cell quiescence [85]. Biguanides administered with olaparib inhibit SNAIL and consequent drug‐resistant ovarian cancer cell tumorigenesis [62]. TWIST mediates the paclitaxel‐resistance of gastric cancer cells [86].

An additional example of tumor plasticity induction is the GATA6 epithelial master regulator in pancreatic ductal adenocarcinoma. Loss of GATA6 with the concomitant loss of HNF1A and HNF4A leads to the development of tumor plasticity, evasive phenotype, and lung metastases [87].

#### 3.1.3 Therapeutic Possibilities Offered by Plastic Cancer Networks

Master regulators of cancer cell plasticity are listed in Section 3.1.2. already provided several examples of promising therapies that suppress plastic cancer cells by inhibiting HOXA5, SMAD4, and SNAIL [62, 64, 65]. An additional important therapeutic option is to block plasticity‐related signaling pathways, such as the NOTCH pathway. Dysregulation of the NOTCH pathway promotes epithelial–mesenchymal transition, metastasis, the development of cancer stem cells, and drug resistance by inducing cellular plasticity [66, 88]. There are several clinical trials involving inhibitors of gamma‐secretase and ADAM (both NOTCH pathway activator proteases); NOTCH itself; or the NOTCH receptor ligands, DLL3 or DLL4 [66]. Alternative pathways are the usual escape routes of cancer cells [69, 89]. The HIPPO pathway may serve as another escape route [89] using cell plasticity. YAP and TEAD, two members of the HIPPO pathway, were shown to engage the epithelial–mesenchymal transition transcription factor SLUG (detailed in Section 3.2) to repress apoptosis. Pharmacological co‐inhibition of YAP and TEAD enhanced EGFR/MEK inhibition‐induced cancer cell apoptosis [90].

Sometimes, quite indirect routes can also be successful in combating cancer cell plasticity. For example, targeting the mevalonate pathway overcomes PLK1 inhibitor resistance of colorectal cancer cells [61]. Inhibition of the mevalonate pathway impairs the synthesis of dolichol. Dolichol is a key factor in the glycosylation‐mediated activation of the AXL receptor. Thus, impairing the mevalonate pathway provides an additional, upstream inhibition of the GAS6/AXL‐PLK1‐TWIST pathway, a key mediator of tumor progression, metastasis, and therapy resistance [61].

**FIGURE 2:**
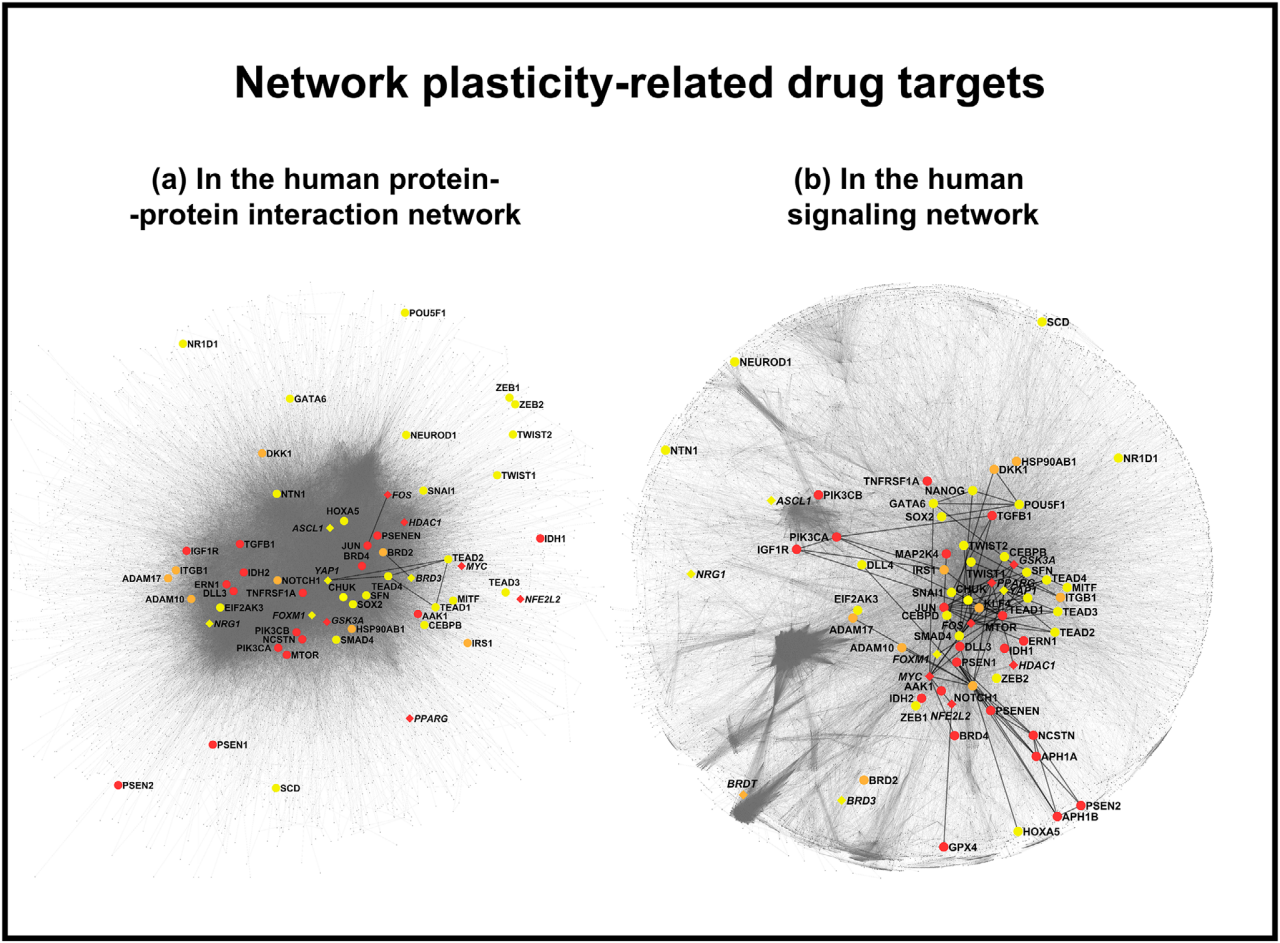
This figure shows the position of cancer network plasticity‐related drug targets in a) the human protein–protein interaction network and b) the human signaling network. Drug targets mentioned in Sections 3.1–3.5 are listed in Table 1. The following isoforms and subunits of drug targets were also visualized using the HGNC symbol system, such as ZEB1‐2, TWIST1‐2, NOTCH1, gamma‐secretase subunits PSEN1‐2/NCSTN/PSENEN/APH1A‐B, ADAM10 and ‐17, TEAD1‐4, PPARG, IRE‐alpha = ERN1; MKK4 = MAP2K4, OCT4 = POU5F1, TGFB1, PI3K = PIK3CA‐B, NRF2 = NFE2L2, PERK = EIF2AK3. Red: 26 drug target isoforms and subunits with approved drugs. Orange: 10 drug target isoforms and subunits with investigational drugs. Yellow: 31 drug target isoform and subunit candidates. Diamonds and *italic protein names* denote IDPs. Network data were downloaded from the databases BioPlex 3.0 (HEK293T cell line data) [91] and SIGNOR 4.0 [92] and were visualized using the OpenOrd and subsequent Yifan Hu layout algorithms of Gephi [93].

Figure 2 shows the position of cancer network plasticity‐related drug targets in the a.) human protein–protein interaction network; b.) human signaling network [91, 92, 93, 94]. The signaling network is more modularized than the protein–protein interaction network, which is what we expect from the larger segregation of signaling pathways. Visual arrangement of approved or investigational drug targets, as well as drug target candidates, shows no particular preference for certain network modules in both networks. Similarly, IDP drug targets or target candidates are also scattered in the network, having no particular preference for a visual position. This arrangement reinforces the view that the development and maintenance of network plasticity involve the entire human molecular network. Target candidates are slightly more peripheral in the protein–protein interaction network than in the signaling network. This is what we would expect, since signal propagation does not need a high density of protein–protein interactions. The significantly less direct connections between network plasticity‐related drug targets (or target candidates) in the protein–protein interaction network than in the signaling network (Figure 2) suggest that the direct signaling connections between these proteins are mostly transient or causational, like phosphorylation or transcriptional activation.

### 3.2 Plasticity of the Epithelial–Mesenchymal Transition Networks

The epithelial–mesenchymal transition (EMT) plays a crucial role in the release, invasion, and metastasis of cancer cells [2, 3, 95]. EMT becomes enhanced by the hypoxic tumor environment, which results in the protection from anoikis [96]. Intercellular communication of the heterogeneous tumor environment both drives and maintains EMT [97]. First, we describe the plentitude of plastic EMT transitions. Next, we outline the signaling network of EMT plasticity. Finally, we list the therapeutic options related to EMT plasticity.

#### 3.2.1 EMT Plasticity Drives Various Transitions in Cell Phenotype

**FIGURE 3:**
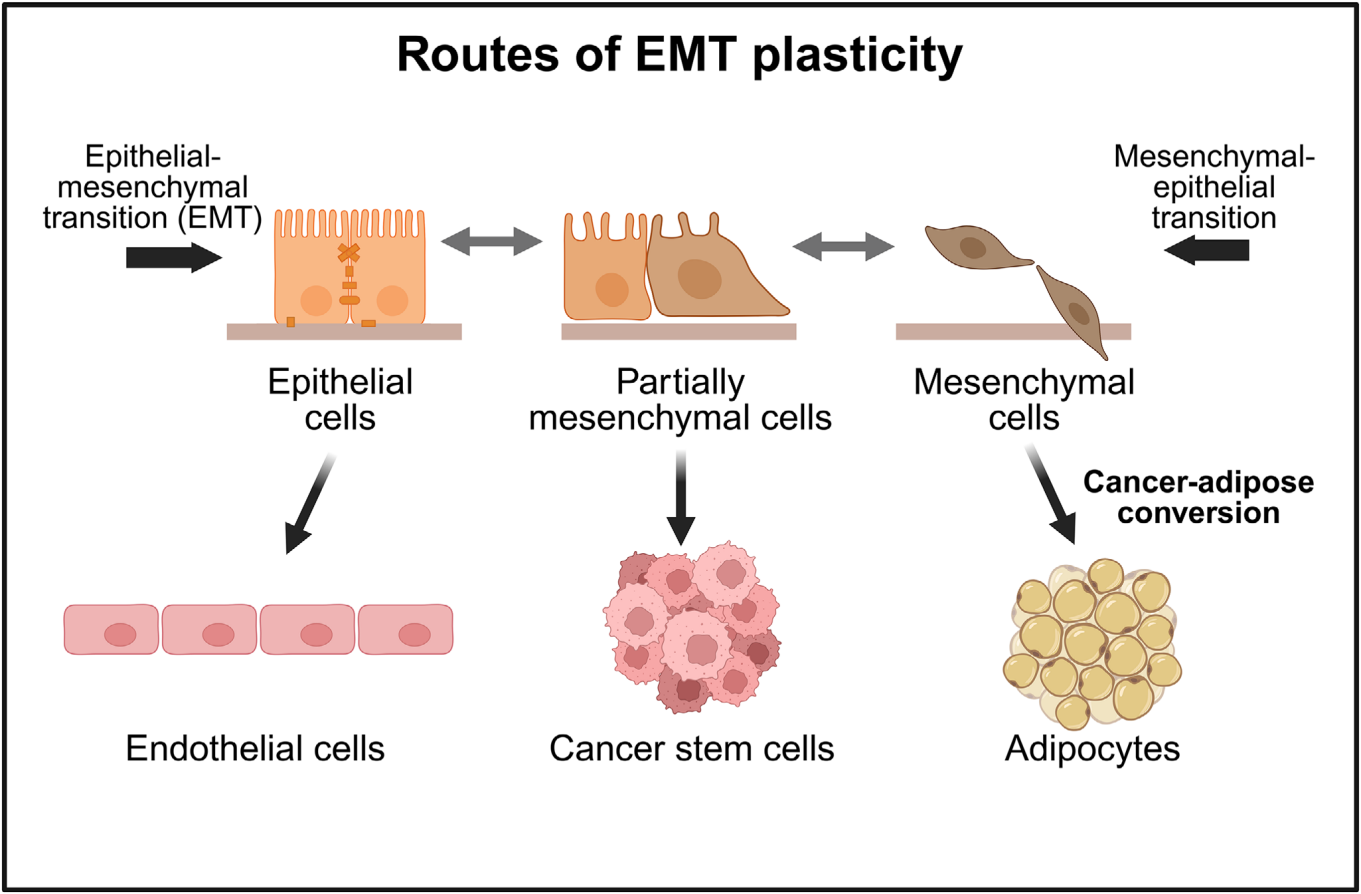
Routes enabled by epithelial–mesenchymal transition plasticity. Created in Biorender.

EMT does not proceed only from an epithelial (tissue‐like) to a mesenchymal (invasive type) state but can also be reversed to a mesenchymal–epithelial transition, often needed to complete metastasis [52, 98]. EMT usually displays several intermediate states (showing partial EMT). These intermediate states are often more carcinogenic, metastatic, and drug‐resistant than the mesenchymal state [6, 52, 95, 98, 99, 100, 101, 102, 103, 104, 105, 106, 107]. EMT (and especially its reverse, the mesenchymal–epithelial transition) can proceed towards the cancer stem cell state [89, 108, 109]. The extremely large plasticity of cancer stem cells will be summarized in Section 3.3. The epithelial state may also shift towards an endothelial state, helping the angiogenesis in melanoma and many other carcinomas [3]. The duodenal epithelium may offer a protective niche to pancreatic ductal adenocarcinoma cells that adopt the mimicry of intestinal epithelial cells [110]. EMT can also be directed towards the differentiated adipocyte state (Figure 3). This cancer‐adipose conversion provides important, novel combination therapy options of ZEB1 or MDM2 activators together with Rosiglitazone [111]. As an additional sign of adaptation‐directed EMT plasticity, EMT involves several key players of the cellular learning process [13].

#### 3.2.2 Signaling Network of EMT Plasticity

EMT signaling involves several hundred nodes. First, we list some key actors mediating EMT plasticity. Next, we summarize key results of EMT signaling network studies, which reveal mechanisms of EMT plasticity. Finally, we show the important role of non‐coding RNAs and chromatin modifications in EMT plasticity.

TGF‐beta is a key activator of EMT. The cell membrane protein CD147 induced the dedifferentiation and malignancy of hepatocellular carcinoma cells via the activation of matrix metalloproteinases, which activated TGF‐beta. Importantly, all‐*trans*‐retinoic acid treatment induced the downregulation of CD147 and reversed these effects [112]. The master transcription factor families ZEB, SNAIL, and TWIST emerged as key factors of EMT plasticity [2].

Positive feedback loops within the EMT signaling network were shown to increase EMT plasticity in the complex study by Hari et al. [49] using discrete, parameter‐independent (Boolean) and continuous, parameter‐agnostic (RACIPE) models. This study paved the way for novel therapeutic interventions (e.g., edgetic therapies [113]) that break the positive feedback loops of EMT signaling networks [49]. Mathematical modeling of single‐cell RNA sequencing data dynamics in EMT highlighted SFN (stratifin, important in the maintenance of epithelial polarity, as well as in the G2/M cell cycle checkpoint) and NRG1 (neuregulin1, an ErbB receptor family protein having a key role in synaptic plasticity) as key regulators of plastic, intermediate EMT states [114]. A large‐scale protein kinase interactome profiling study of 18 diverse cancer cell lines revealed that EMT plasticity‐altered endocytic and vesicle trafficking pathways were controlled by the AAK1 (adapter‐associated kinase 1) interaction network [48]. AAK1 emerges as a promising drug target alone or in combination with HDAC inhibition [115].

The EMT signaling network is modulated by various non‐coding RNAs and epigenetic modifications. As an example of these, a large set of microRNAs helps the mediation of cross‐talk between TGF‐beta, NOTCH, and WNT in EMT [108]. Mutually inhibitory feedback loops involving mir34/SNAIL and mir200/ZEB are central motifs in EMT cell decision and plasticity [100]. Mir7974 was recently shown to regulate EMT plasticity, promoting a rapidly proliferating epithelial phenotype and reducing the metastatic potential of colorectal cancer [116]. Similarly, circular RNAs also play a central role in EMT‐modulated cancer cell plasticity, tumorigenesis, metastasis, and the development of drug resistance [117]. As an example of epigenetic modifications, the key epithelial marker CDH1 (E‐cadherin) becomes repressed during EMT through increased DNA methylation in its promoter and through the alteration in histone modifications from activation to inhibition. Several other epigenetic modifications also contribute to EMT plasticity [52].

#### 3.2.3 Therapeutic Targets Modulating EMT Plasticity

Promising EMT‐related therapeutic targets are members of the TGF‐beta, WNT, and NOTCH pathways [95, 118, 119]. Recently, targeting the embryonic cell guiding protein netrin‐1 (NTN1) was shown to block metastatic progression of squamous cell carcinoma and endometrial cancer by shifting tumors towards a more epithelial phenotype [120, 121]. The mesenchymal state makes pancreatic ductal adenocarcinoma cells sensitive towards inhibitors of the IRE‐alpha–MKK4 arm of the endoplasmic reticulum stress pathway [122]. Similarly, the frequently observed loss of KDM6A, an X‐chromosome‐encoded histone demethylase (a member of the COMPASS‐like complex), rendered the quasi‐mesenchymal, squamous‐like, metastatic pancreatic cancer cells sensitive to BET inhibitors, which reversed squamous differentiation and restrained tumor growth, reopening the window for traditional therapies [123]. The mesenchymal state predisposes cells to ferroptosis, a cell death pathway mediated by iron‐dependent phospholipid peroxidation. The recent study of Schwab et al. [124] showed that in cancers overexpressing ZEB1 (a key mediator of EMT plasticity), the combinatorial therapy of SCD (stearoyl‐CoA‐desaturase, an enzyme protecting cells against ferroptosis by increasing their polyunsaturated fatty acid content) and GPX4 (glutathione‐peroxidase 4, an enzyme protecting cells against oxidation occurring in ferroptosis) inhibitors has a high therapeutic promise. These studies opened new therapeutic possibilities for targeting metastasis‐prone mesenchymal cancer cells or for directing them towards the endothelial state.

### 3.3 An Extreme Case of Network Plasticity: Cancer Stem Cells

Cancer stem cells are a minority subset of cancer cells that survive conventional chemotherapies, repeatedly renew tumors, and drive tumor metastasis progression by slowing down their own proliferation rate [125], by building a supportive niche, and by utilizing the extreme plasticity in their phenotype, signaling, and metabolism [109, 126, 127, 128, 129]. Already, early‐stage metastatic breast cancer cells possessed a distinct stem cell‐like gene expression signature together with EMT, pro‐survival, and dormancy‐associated genes [99]. Actual properties of cancer stem cells depend on the individual “stress‐history” of the given tumor [126]. Cancer stem cells also increase the plasticity of their neighbors, such as that of tumor‐associated macrophages [130]. By increasing the plasticity of their neighborhood, cancer cells transform their surrounding environment into the supportive niche mentioned before (Figure 3) [128, 129, 130].

EMT may also lead to cancer stem cell formation [99, 108, 128]. Cancer stem cells have an extremely plastic signaling network (where even the level of plasticity may rapidly change upon changes in the environment) [126]. Not surprisingly, their modeling was called “modeling mayhem” [6]. NANOG, SOX2, OCT4, KLF4, MYC, WNT, TGF‐beta, as well as Notch and Hedgehog pathway members emerged as key modulators of stemness. The majority of this list overlaps with key players of EMT [6, 126].

Differentiation therapy was first suggested by Stuart Kauffman in 1971 [131] and is increasingly used to induce the differentiation of cancer stem cells [3, 132, 133]. A conventional therapy is the treatment of acute myeloid leukemia by all‐*trans*‐retinoic acid [3]. Inhibitors of isocitrate dehydrogenase (IDH1 and IDH2), such as ivosidenib, enasidenib, or vorasidenib, are approved drugs that induce the differentiation of cancer stem cells in *IDH1/2*‐mutated acute myeloid leukemia or glioma, respectively [134]. Ferroptosis, an iron‐dependent oxidative form of programmed cell death, has emerged as a potential focal point in the eradication of cancer stem cells and in tumor differentiation [135]. The ionophore salinomycin killed breast cancer stem cells in mice at least a hundred times more effectively than the anticancer drug paclitaxel and induced tumor differentiation to epithelial cells [136]. Salinomycin induces lysosomal iron sequestration, leading to the production of reactive oxygen species, lysosome membrane permeabilization, and ferroptosis [137]. DKK1 (Dickkopf WNT signaling pathway inhibitor 1) increased the expression of SLC7A11 sodium‐independent cystine–glutamate antiporter, which protected metastasizing cancer stem cells from ferroptosis. Combined treatment of a ferroptosis inducer (Erastin or LSR3) and DKK1 inhibitor (WAY262611 or Gallocyanine) exhibited a synergistic effect, inhibiting metastasis [138]. We have mentioned combination therapies of ZEB1 or MDM2 activators together with Rosiglitazone that induce cancer‐adipocyte differentiation (competing with cancer stem cell formation) in Section 3.2.1 [111]. However, PPAR‐gamma has recently been shown to induce the retrodifferentiation of hepatocellular carcinomas [139], which warrants its careful, selective use as a differentiation agent. Activation of G‐protein‐coupled estrogen receptor signaling shifted melanoma stem cells to a more differentiated and more drug‐sensitive state [140]. Differentiation therapy is a typical example of a therapeutic option based on cancer cell plasticity. The above studies provided novel routes to expand the traditional, well‐established ways of differentiation therapy of acute myeloid leukemia towards cancer stem cell differentiation.

### 3.4 Network Plasticity Changes in Cancer Drug Resistance Development

**FIGURE 4:**
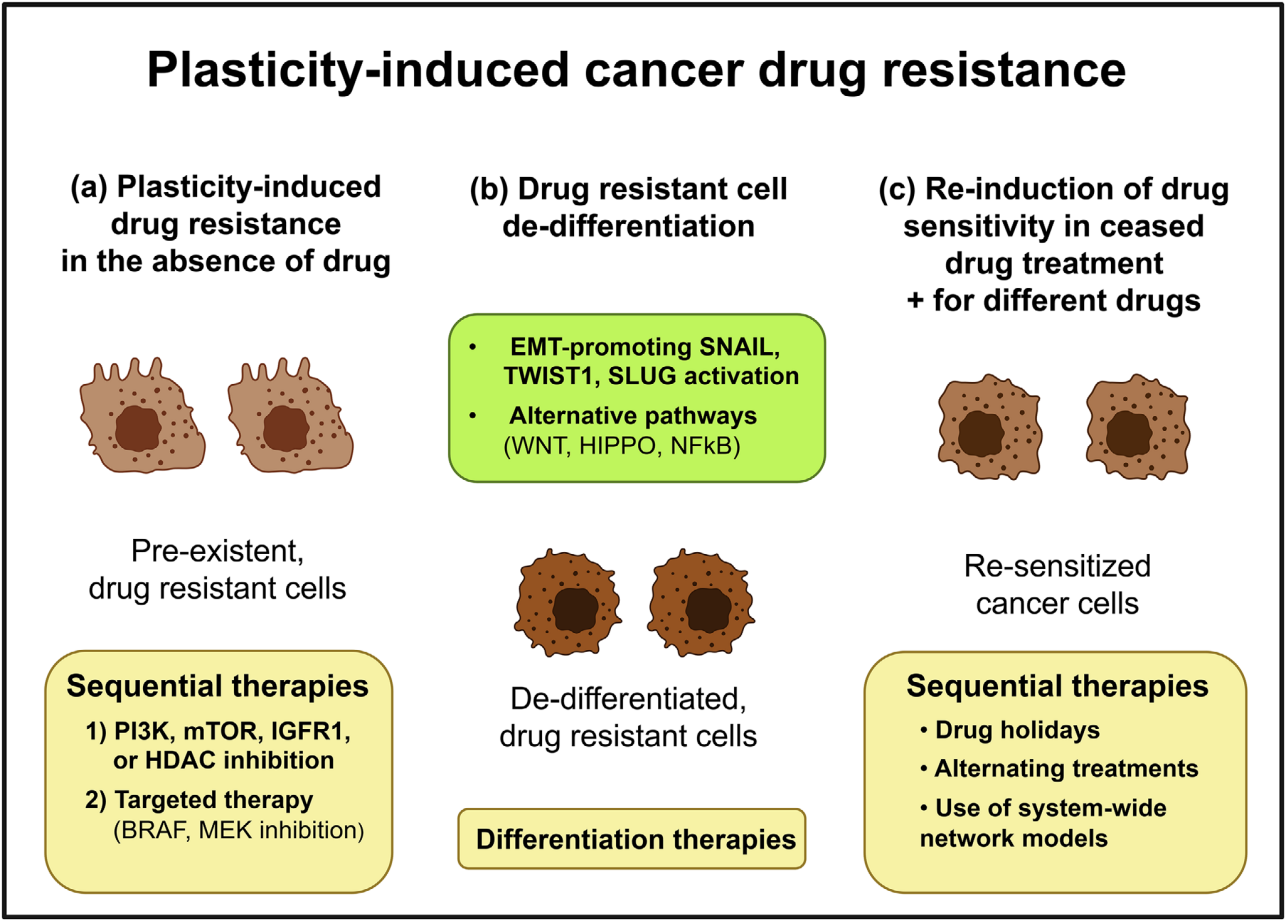
Cancer drug resistance: a learning process of plasticity changes. Created in Biorender.

The development of cancer drug resistance was recently described as a cellular learning process (Figure 4) [14, 15, 53]. Increase in cancer cell plasticity (such as that of cells undergoing partial EMT or cancer stem cells described in Sections 3.1–3.3) induces drug resistance [6, 52, 95, 98, 99, 100, 101]. This process, together with the well‐documented cancer cell plasticity increase by drug treatment [7], forms a vicious cycle of self‐perpetuating difficulty.

#### 3.4.1 Plasticity‐Induced Pre‐Existent Drug‐Resistant Cancer Cells: Therapy Options

The plasticity of the cancer cell phenotype permits the emergence of drug resistance even in the absence of drug treatment. This intrinsic drug resistance characterizes only a rare subpopulation of cancer cells (less than 0.1% of cells). This transient, drug‐resistant state may become stabilized and persist through several generations of dividing cancer cells (Figure 4) [141, 142, 143]. Long‐lived, rare but coordinated fluctuations in gene expression are major sources of pre‐existent drug‐resistant cells. These and other sources of noise are crucial in raising cellular heterogeneity (including pre‐existent drug‐resistant cells) in cancer cell populations [141, 144, 145]. These pre‐existent drug‐resistant cells have been shown to often become dormant, reducing their proliferation rate, which helps their initial escape from several anticancer drugs targeting cell division mechanisms [14]. However, sequential therapies, e.g., targeting first by a “cellular memory drug” (i.e., a drug that affects cellular memory formation or cellular forgetting mechanisms) [33, 53] such as PI3K, mTOR, IGFR1, or HDAC inhibitors, followed by a targeted therapy (e.g., that of BRAF or MEK inhibitors) proved to be useful to sensitize cancer cells to targeted therapy by previous eradication of plasticity‐induced, pre‐existent drug‐resistant cells [7, 141, 144, 145]. Sometimes this strategy may work indirectly. As an example, histone deacetylase (HDAC) inhibition cannot directly eradicate drug‐resistant neuroblastoma cells. However, HDAC inhibition improves the response of consecutive pro‐apoptotic drugs by restoring apoptosis‐promoting JNK activity [145].

#### 3.4.2 Plasticity of Drug‐Resistant Cancer Cell Networks: Therapy Options

Intermodular hubs of cancer cell protein–protein interaction networks often contain signaling domains playing a key role in cancer signaling. These interactome module bridges proved to be important in breast cancer prognosis [67]. Crucially, signaling pathway cross‐talks may offer novel drug combinations [69]. Signaling pathways independently acting on the same target (such as ERK and YAP1 regulating cell cycle control) significantly contribute to cell plasticity and can substitute for each other in the development of drug resistance [89]. In breast cancer cells treated with protein kinase/phosphatase inhibitors, the signaling network has been shown to become heterogeneously resistant to the drug, which shows an important sign of network plasticity [47]. Importantly, network plasticity and the relatively high noise of cancer cells themselves re‐induce the drug‐dependent state if the drug administration is ceased. This is the reason why re‐sensitization to a repeated drug challenge occurs, and why “drug holiday” has become an important treatment option for cancer patients [146, 147].

Several forms of therapy resistance are achieved by cancer cell dedifferentiation. This process often involves the EMT promoting SNAIL, TWIST1, and SLUG. SNAIL and TWIST1 activate PERK kinase and its target, NRF2. NRF2 is a master transcription factor of the antioxidant response, a key regulator of therapy resistance [2]. The other EMT‐related protein, SLUG, promotes cell survival by repressing the pro‐apoptotic protein PUMA [2]. Activation of other alternative pathway kinases, such as the WNT pathway kinase GSK3, several HIPPO pathway kinases, and the NFkB‐activating kinase CHUK, has also been observed [48].

Importantly, as cancer cells increase their plasticity and escape from the lethal consequences of a drug treatment, developing resistance, they become sensitive to other treatments. For example, EGFR inhibitor‐resistant cells are sensitized to the genotoxic drug gemcitabine [3]. This opens the way for sequential therapies, where, after a shorter treatment window, the treatment rapidly shifts to a completely different mode of attack. Such sequential therapies were already mentioned in Section 3.4.1. where therapies with “cellular memory drugs” sensitized cancer cells to targeted therapies by eradicating the minor, pre‐existent drug‐resistant cell population. The prediction of biomarkers and protocols of such sequential therapies is an important promise of recently developed system‐wide network models [148].

### 3.5 Therapeutic Options Offered by Modulation of Network Plasticity in Cancer

Table 1 summarizes the plasticity‐modulating therapeutic options mentioned in Section 3. For an extensive coverage of therapy options for overcoming tumor cell plasticity‐induced drug resistance, see Shi et al. [7]. Importantly, 12 out of the 55 drug targets (22%) listed in Table 1 are intrinsically disordered proteins with a higher disorder content than 20% [42]. This highlights the important role of molecular‐level plasticity in the development of plastic phenotypes in cancer. Table 1 lists 20 drug targets with more than 70 approved drugs [149]. Additionally, 35 drug targets (see their names in italic) have no approved drugs yet. 9 of them (ITGB1, NOTCH, ADAM, IRS1, HSP90‐beta, BRD2, BRDT, KLF4, and DKK1) had investigational phase drugs (Bexotegast, Aderbasib/Aprastat/XL787, Crenigacestat, NT‐219, Zelavespib, Birabresib, JQ1, APTO‐253, and WAY 262611/Gallocyanine, respectively). This list convincingly demonstrates that network plasticity opens new areas in drug development against cancer.

**TABLE 1:** Cancer cell plasticity‐related drug targets.^a^

Drug target ^b^	Involvement in cancer cell plasticity	Drug(s)^c^	Refs.
*HOXA5, SMAD4, MITF*, JUN, FOS^d^ *ASCL1* ^d^ *NEUROD1*, *SNAIL (SNA1)*, *ZEB, TWIST*, *GATA6, FOXM1* ^d^ *, CEBPB, CEBPD, REV‐ERB‐alpha, INSM1*	Master transcription factor of cancer plasticity	Adapalene, Irbesartan, Vinblastine, Nadroparin	[2, 59, 62, 63, 64, 65, 78, 80, 81, 82, 83, 86, 87]
TNFR1, *ITGB1*	Glioblastoma mesenchymal transition activator	Tasonermin	[83]
*NOTCH*, gamma‐secretase, *ADAM*, DLL3/*DLL4*	NOTCH cancer plasticity pathway receptor, activator, and ligand	Nirogacestat, Tarlatamab	[66, 88]
*YAP1* ^d^, *TEAD*, CHUK	HIPPO cancer plasticity pathway members	Acetylcysteine, Aminosalicylic acid, Mesalazine, Sulfasalazine	[48, 90]
*IRS1, HSP90‐beta*	Plastic network module connector		[32, 67, 73]
PPAR‐gamma^d^	Inducer of cancer‐adipocyte differentiation	∼25 approved drugs	[111]
*SFN*, *NRG1* ^d^	Regulator of partial EMT		[114]
*NTN1*	Cell guidance in partial EMT		[120, 121]
AAK1, IRE‐alpha–MKK4	EMT plasticity regulator	Fostamatinib, Encorafenib	[48, 115, 122]
*BRD2*, *BRD3* ^d^, BRD4 and *BRDT* ^d^	Chromatin plasticity regulators in EMT	Colchiceine	[123]
*SCD*/GPX4	Ferroptosis in EMT	Glutathione	[124]
*NANOG*, *SOX2*, *OCT4*, *KLF4*, MYC^d^, TGF‐beta	Cancer stem cell plasticity modulator	Acetylsalicylic acid, Dimethyl sulfoxide, Doconexent, Nadroparin, Pirfenidone, Terazosin	[6, 127]
*DKK1*	Cancer stem cell ferroptosis		[138]
IDH1 and IDH2	Cancer cell dedifferentiation in mutants	Indocyanine green, Ivosidenib, Enasidenib, Olutasidenib, Vorasidenib	[134]
PI3K, mTOR, IGF1R, HDAC1^d^	Cellular memory (or forgetting) related protein	Alpelisib, Copanlisib, Duvelisib, Idelalisib, Leniolisib, Everolimus, Pimecrolimus, Sirolimus, Temsirolimus, Brigatinib, Fostamatinib, Glasdegib, Fingolimod, Givinostat, Panobinostat, Phenylbutyric acid, Romidepsin, Vorinostat – followed by conventional targeted therapy	[7, 33, 53, 124, 144, 145]
NRF2^d^, *PERK*	Antioxidant plasticity master transcription factor and its activator	Carbocisteine, Ethionamide, Omaveloxolone	[2]
GSK3^d^	Member of the alternative WNT pathway often induced by cancer drug resistance	Fostamatinib	[48]

^a^ The network representation of drug targets is shown on Figure 2.

^b^ Targeting drugs were identified using DrugBank [149].

^c^ Italic: targets with no approved drugs yet. We note that some key master transcription factors may also be targeted via their protein neighbors [26].

^d^ Intrinsically disordered proteins were identified by DisProt using 20% disorder content as a threshold [42].

## 4 Modulation of Network Plasticity in Diabetes

Network plasticity plays a key role in emerging therapies that delay, prevent, or reverse the onset of the diabetic state. First, we introduce the key players involved in network plasticity in type 1 and type 2 diabetes. Next, we provide examples of how network plasticity contributes to the development of diabetes. We show how vascular smooth muscle cell plasticity contributes to diabetes complications. Last, we summarize three ways of modulating cell plasticity to achieve beta cell repopulation: 1) beta cell regeneration; 2) transdifferentiation to beta cells; and 3) proliferation of usually quiescent beta cells.

### 4.1 Key Factors of Network Plasticity in Type 1 and Type 2 Diabetes

In early‐onset type 1 diabetes, the insulin secretion of pancreatic beta cells becomes impaired. FOXO1 deficiency induces beta cell dedifferentiation, resulting in a more plastic, progenitor‐type beta cell phenotype that expresses NRG3, OCT4, NANOG, and L‐MYC [150]. Dedifferentiation of beta cells is driven by hyperglycemia‐enhanced endoplasmic reticulum stress, where the unfolded protein response drivers, XBP1, IRE1, PERK, ATF6, and CHOP play a key role [21, 151, 152, 153]. Beta cells may also transdifferentiate into other cell types, such as pancreatic alpha cells [21, 154]. Simultaneous inhibition of EIF5A and NOTCH pathways was suggested to upregulate immune tolerance and differentiation of cytotoxic T cells, which kill pancreatic beta cells, restoring beta cell dysfunction. Such a therapeutic modality may also be used following islet cell transplantation or after adoptive cell transfer [155]. Deletion of XBP1 in beta cells of non‐obese diabetic mice led to protection against the onset of islet inflammation and consequent insulitis. This was achieved by reducing autoantigens of beta cells, potentially involving the PREB, NME2, HNF6, NRF3, and ASF1A proteins [156].

Type 2 diabetes is the most prevalent form of diabetes, characterized by insulin resistance and relative insulin deficiency. As a sign of protein–protein interaction network plasticity, intermodular proteins play a key role in the regulation of type 2 diabetes. Importantly, proteins that link diabetes and heart disease overlap with those that link heart disease and obesity [157]. The master transcription factor of cell plasticity HOXA5 is also involved in the development of obesity and type 2 diabetes [158]. Another cell plasticity master transcription factor, SMAD4, promotes diabetic nephropathy [159].

### 4.2 Network Plasticity Helps the Progression to the Diabetic State

We highlight the contribution of network plasticity to diabetes development by two examples: 1) Network plasticity‐derived differences in the effects of acute versus long‐term high glucose and insulin receptor inhibition, where long‐term treatment mimics conditions of the diabetic state. 2) Endoplasmic reticulum stress inhibitors—as an example of network plasticity modulation helping to recover pancreatic beta cells from long‐term stress conditions.

When pancreatic islets were incubated in the presence of high glucose, they exhibited a stronger response after 3 h, which then developed into glucose insensitivity after 6 h [160]. Similarly, hyperglycemia led to the PKC beta II‐induced phosphorylation and mitochondrial translocation of p66SHC, which in turn induced the production of reactive oxygen species. After normoglycemia had been restored, p66SHC remained in the mitochondria, resulting in a hyperglycemic molecular memory in human aortic endothelial cells. However, persistent hyperglycemia upregulated reactive oxygen species, which led to increased PKC beta II and p66SHC levels, creating a vicious cycle [161]. A similar, biphasic effect was observed when 3T3‐L1 cultured fat cells were exposed to insulin receptor auto‐antibodies. Acute administration provoked a more efficient deoxyglucose uptake, while prolonged incubation led to insulin insensitivity [162]. These biphasic responses are clear signs of network plasticity modulating cellular learning [12, 13, 14, 15, 16, 17, 18] and forgetting processes [33] involved in diabetes development.

Inhibitors of beta cell endoplasmic reticulum stress, such as many of the approved antidiabetic drugs, thiazolidinediones, GLP1 agonists, resveratrol, or tauroursodeoxycholic acid, emerge as key factors preventing or delaying diabetes. However, the beta cell bioavailability of these stress inhibitors is typically rather poor, which necessitates the use of specifically targeted delivery options [163, 164]. Modulation of endoplasmic reticulum stress or the mTOR pathway may also prevent or delay the onset of diabetes [21, 163]. In addition, inhibitors of polyamine biosynthesis, such as difluoromethylornithine, also enhance beta cell regeneration [21].

### 4.3 Involvement of Vascular Smooth Muscle Plasticity in Diabetes Complications

Vascular smooth muscle cell plasticity is a key driver for several macrovascular complications associated with type 2 diabetes. Vascular smooth muscle cells may undergo phenotypic transformation towards increased proliferative, inflammatory (macrophage‐like), or osteogenic (bone‐like) properties. Pro‐ and antiatherogenic adipokines, such as leptin and retinol binding protein 4, as well as adiponectin and omentin 1, govern these processes. Hyperglycaemia and insulin deficiency are well‐known factors contributing to vascular smooth muscle cell calcification. Vascular smooth muscle cell dysfunction involves PI3K/AKT, MAPK, AMPK/mTORC, and BMP2 signaling [164].

### 4.4 Pancreatic Beta Cell Plasticity Has a Key Role in Diabetes

Beta cell plasticity is driven by a large number of transcription factors, including ATF4, FOXA2, FOXO1, GATA6, GLIS3, HES1, HNF1A, HNF4, ISL1, LDB1, MAFA, MAFB, MNX1, NEUROD1, NGN3, NKX2, NKX6, NRX, PAX4, PAX6, PDX1, SOX9, STAT3, and ZEB [21, 154]. Only three transcription factors of this list (GATA6, NEUROD1, and ZEB) have been associated with cancer cell plasticity (see Table 1). The mTOR signaling pathway (including GRB10, RPTOR, and RICTOR) is crucial for beta cell maturation [21]. Signaling pathway plasticity is often increased by noncoding RNAs. Gojani et al. [21]. list ∼30 microRNAs and ∼20 long noncoding RNAs involved in beta cell plasticity.

### 4.5 Modulation of Beta Cell Plasticity 1: Beta Cell Regeneration

Beta cell regeneration emerged as a potential therapy for diabetes [21]. Transient treatment of pancreatic ductal progenitor cells from juvenile and adult type 1 diabetes patients by the FDA‐approved EZH2 inhibitors, GSK126 and Tazemetostat, induced a shift of progenitor cells towards pancreatic beta cells [163]. Beta cell‐derived progenitor cells were redifferentiated upon the addition of the “redifferentiation cocktail” containing soluble nicotinamide, exendin‐4, activin A, and high glucose [154].

### 4.6 Modulation of Beta Cell Plasticity 2: Transdifferentiation to Beta Cells

Transdifferentiation of other cells into beta cells is another widely studied option. Pancreatic alpha cell transdifferentiation into beta cells is a natural route of beta cell regeneration. This process can be enhanced by glucagon receptor antibodies. The process is mediated by FGF21 and glucagon‐like peptide 1 (GLP1) secretion [21]. Overexpression of several beta cell plasticity‐increasing transcription factors (listed above), such as MAFA, NGN3, PAX4, PAX6, and PDX1, prompted their examination as candidates for potential targeted expression therapy [153]. Adipose‐derived mesenchymal stem cells can transdifferentiate into beta cells. This process is modulated by the upregulation and downregulation of FOXO1 in early and later phases of transdifferentiation, respectively [21]. Bone marrow‐derived mesenchymal stem cells were transdifferentiated into beta cells using the “transdifferentiation cocktail” of high glucose, nicotinamide, beta‐mercaptoethanol, betacellulin, and IGF1 [21]. Human umbilical cord mesenchymal stem cells were shown to reverse beta‐cell dedifferentiation. This reversion was facilitated by the secretion of an interleukin receptor antagonist by stem cells [21].

### 4.7 Modulation of Beta Cell Plasticity 3: Beta Cell Proliferation

Beta cells are usually quiescent. Their proliferation can be stimulated by inhibiting the DYRK1A kinase. Fostamatinib is an inhibitor of DYRK1A kinase approved to treat rheumatoid arthritis [21, 132]. Combining a DYRK1A inhibitor with a glucagon‐like peptide 1 receptor agonist accelerates beta cell proliferation [21]. These numerous examples demonstrate the rich repertoire offered by beta cell plasticity for anti‐diabetic therapies against both type 1 and type 2 diabetes.

## 5 Modulation of Network Plasticity in Neurodegeneration

Despite the importance of cell and cellular network reconfigurations in neurodegeneration [165], there are only a few studies on network plasticity in these diseases. Proteins of the periphery of gene regulatory networks were shown to have preferential contribution to the development of Alzheimer's and Parkinson's disease [50, 51]. Importantly, known Alzheimer's disease genes were shown to be in the periphery of gene regulatory networks and to be connected to network hubs [51]. This is a typical network configuration that increases network plasticity [10].

### 5.1 Key Network Plasticity‐Related Players of Neurodegenerative Disease Progression

Several proteins emerged as important hubs of plasticity‐related neurodegenerative disease progression. Histone H1 is a key modulator of chromatin plasticity. Disregulation of histone H1 expression, localization, and posttranslational modifications has been shown to contribute to neurodegenerative diseases [166]. Disease‐associated changes of the key antioxidant pathway NRF2/KEAP1 are involved in the pathogenesis of Alzheimer's disease, Parkinson's disease, Huntington's disease, and amyotrophic lateral sclerosis, leading to protein aggregation, mitochondrial dysfunction, and neuroinflammation [167]. Alzheimer's disease‐associated posttranslational modifications transform several molecular chaperones to epichaperones, i.e., multimeric scaffolds that rewire protein–protein interaction networks [73]. An increase in presynaptic plasticity is associated with exosomal protein function and may significantly compensate for initial neurodegeneration [168].

Recently, several microRNAs have been shown to play key roles in the development of neurodegenerative diseases. A brain‐enriched microRNA, mir137, modulates amyloid‐beta production, tau phosphorylation, synaptic plasticity, and neuroinflammation, preserves mitochondrial function, and mitigates oxidative stress. The involvement of mir137 in the development of Alzheimer's disease posits this microRNA as a potential biomarker and drug target candidate [169]. Mir146a‐5p is a potent anti‐inflammatory agent. Network analysis indicated a mir146a‐5p‐associated signaling subnetwork involved in Alzheimer's disease pathology [170]. Downregulation of miR17, miR20a, and miR106b was involved in the elevation of thioredoxin‐interacting protein (TXNIP), which activated ASK‐1, JNK, and MAPK, as well as contributed to the misfolding and aggregation of α‐synuclein, a hallmark of Parkinson's disease [171]. Additionally, 25 long noncoding RNAs were identified as participants in an Alzheimer's disease‐associated interaction network of the temporal cortex of 396 postmortem brain RNA‐seq samples [172].

### 5.2 Recent “Plasticity‐Breakthroughs” in Neurodegenerative Disease Clinical Trials

There are several ongoing clinical trials for molecules improving synaptic plasticity in Alzheimer's disease, including a phase II trial of Levetiracetam, an approved drug against epileptic seizures [173, 174]. Additional ∼30 clinical trials in Alzheimer's disease address the inhibition of beta‐amyloid protein or tau protein aggregation, the inhibition of amyloid precursor protein cleaving beta‐secretase (BACE), enhanced beta‐amyloid or tau plaque clearance, and enhanced glial protection, or involve glutamate modulators and acetylcholinesterase inhibitors [175]. The early phase of Parkinson's disease is conventionally treated with dopamine analogues. However, in later phases, the loss of dopamine‐responsive neurons requires regenerative therapies. Restoration of neuronal activity, both in Alzheimer's and Parkinson's disease, increasingly involves stem cell therapy. There are more than a hundred ongoing clinical trials in this area [24].

Recently, breakthroughs in three clinical trials in Alzheimer's disease were reported [176, 177, 178]. A phase randomized, double‐blind, placebo‐controlled IIa trial involved 49 Alzheimer's disease patients, and used specially treated, allogeneic bone marrow‐derived mesenchymal stem cells (laromestrocel/Lomecel‐B), which did not provoke an immune response. The treatment was safe, improved cognitive functions, and showed a potential improvement in brain structure [176]. In Parkinson's disease, a US/Canadian phase I trial used human embryonic stem cell‐derived dopaminergic neurons [178], while a Japanese phase I/II trial used allogeneic induced pluripotent stem cell‐derived dopaminergic progenitors [177]. Both trials showed improvement in motor functions—even as large as 50%. Moreover, there was no incidence of dyskinesia (involuntary movements), which has been a common problem with earlier fetal‐tissue transplants [177, 178]. These studies show a very promising sign of how cellular and network plasticity may be used in regeneration therapies of currently largely untreatable neurodegenerative diseases.

## 6 “Plasticity Drugs” and “Plasticity Therapies”: Research Gaps and Perspectives

This review has convincingly demonstrated that in recent years, a large number of drug targets (see e.g., Table 1) and therapeutic options (such as regeneration therapies) have become available, all of which are based on our increasing understanding of the plasticity of diseased cells and their network representations. We refer to the drugs related to cell plasticity changes as “plasticity drugs” and the therapeutic options utilizing cell plasticity remodeling as “plasticity therapies.”

**FIGURE 5:**
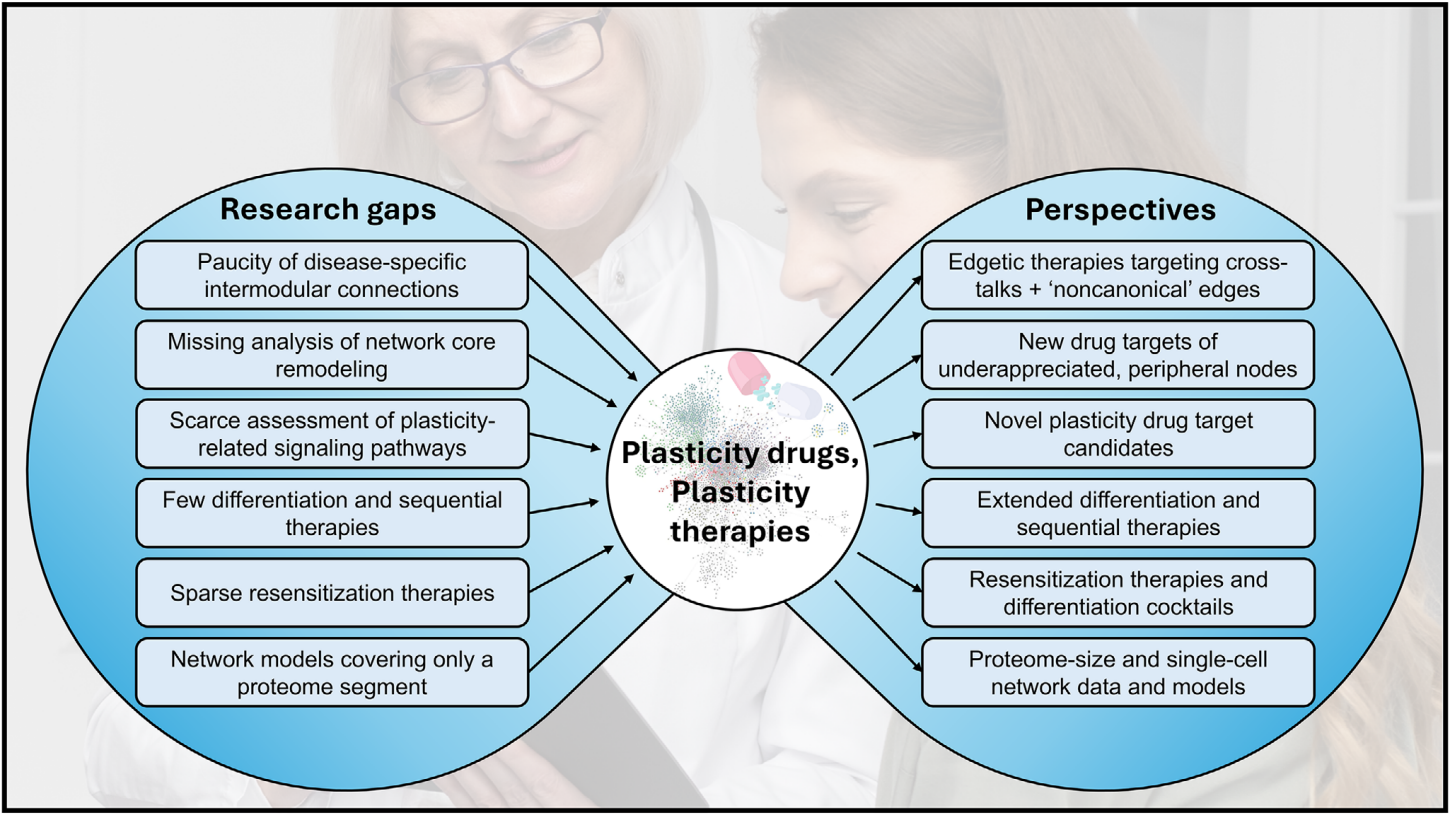
Plasticity drugs and plasticity therapies: research gaps and perspectives. The background image was designed by Freepik.

There are several research areas of plasticity changes in protein–protein interaction, signaling, and gene regulation networks related to cancer, diabetes, and neurodegeneration, which require greater attention (Figure 5).

### 6.1 Disease‐Specific Intermodular Connections in Cancer and Other Diseases

In plastic networks, the boundaries of network modules become “fuzzier” by the emergence of intermodular connections [26, 32]. In agreement with this, an increasing number of studies show the importance of novel, intermodular network connections specifically characterizing the disease state. Most of these connections are provided by novel network edges or by peripheral network proteins [31, 32]. The contribution of intermodular hubs can also be observed. BRAF and IRS1 are classic examples of the intermodular hubs in cancer [25, 67]. In signaling networks, these connections are disease‐specific signaling pathway cross‐talks [5, 22]. Disease‐specific cross‐talks significantly contribute to cancer development [25, 56, 58]. Disease‐specific interactome remodeling has recently explored in diabetes [179] and Alzheimer's disease [180].

Newly formed, disease‐specific, intermodular network edges or nodes often shortcut network regions that were distant in healthy cells. Using this mechanism, these disease‐specific, “non‐canonical” connections may re‐route signaling pathways (see Section 3.1.1) [31, 32]. Reorganization of the molecular chaperone protein–protein interaction network has also been observed in protein‐folding related diseases, such as Alzheimer's disease [180].

Examinations of disease‐specific, intermodular connections raise the possibility of network remodeling‐driven drug combinations [69], such as those inhibiting the RAS‐ERK and PI3K‐mTORC pathways [68]. A typical “non‐canonical” connection of a cancer‐specific receptor tyrosine kinase network was found between ERBB1 and IRS1 [32].

Novel, disease‐specific intermodular connections, as well as other disease‐specific protein–protein interactions, such as those establishing positive feedback loops in epithelial–mesenchymal transition networks involved in cancer metastasis and drug resistance formation [49], may be targeted by novel, edgetic therapies [113]. Edgetic therapy is exemplified by the drug design strategy of chemically induced proximity. These interventions introduce bivalent molecules to cells, binding to two target proteins and building a “molecular glue” between them [181]. Such “glues” may change the phosphorylation status of a protein by gluing it to a protein kinase or phosphatase [182]. Sequestering one of the proteins involved in the novel, disease‐specific “non‐canonical” connections to, e.g., a different subcellular compartment or by binding to a protein mega‐complex, may efficiently restore the disease‐specific reorganization of signaling pathways.

Targeting novel, disease‐specific intermodular connections by methods summarized in this subsection offers novel therapeutic routes to combat diseases, which are different from the traditional therapies targeting specific proteins or signaling pathways. Since intermodular connections play a key role in modulating network plasticity (see Section 2), intermodular connection‐specific interventions also enrich plasticity‐related therapies.

### 6.2 Core–Periphery Network Remodeling in Cancer and Neurodegeneration

The increase in network plasticity often involves the reconfiguration of the network core, which becomes larger and fuzzier due to increased core–periphery connections [10, 26]. In agreement with this, a frequently used mode of network remodeling in a disease state is the disease‐specific connection of peripheral nodes to central network hubs. For example, these peripheral nodes play a prominent role in Alzheimer's and Parkinson's disease development [50, 51]. Regretfully, studies on network core remodeling in cancer development or in neurodegeneration are largely missing. Such studies would open new areas of drug design and therapy development.

### 6.3 Plasticity‐Related Cancer Signaling Pathways

There are several master transcription factors that govern cell plasticity in cancer. Their inhibition already provides important therapeutic options and may further enrich our repertoire in the future (Table 1). Several signaling pathways, such as NOTCH and HIPPO, directly contribute to cancer cell plasticity. Inhibition of NOTCH or its ligand, DLL3, has investigational or approved drugs already (Table 1) [66, 88, 90].

Specific attention is already directed towards the plastic, intermediate states of epithelial–mesenchymal transition. The drug target candidate NRG1 or the drug target AAK are key examples of this type of promising future therapeutic intervention [48, 114, 115].

### 6.4 Cancer Differentiation and Sequential Therapies

Differentiation therapies were first suggested in 1971 [131] and have become well‐established in the treatment of acute myeloid leukemia [3]. However, a large number of recent studies have shown that a much wider application of differentiation therapies might be feasible using cancer adipose differentiation provoked by Rosiglitazone or melanoma stem cell differentiation by G protein‐coupled estrogen signaling as examples [111, 140].

Recently, sequential therapies, targeting cancer cells first by a “cellular memory drug” [53], such as PI3K, mTOR, IGFR1, or HDAC inhibitors, followed by a targeted therapy (e.g., by BRAF or MEK inhibitors) proved to be useful to eradicate pre‐existing drug‐resistant cells first and to kill most of the cancer cells only as a second step [7, 53, 141, 144, 145].

### 6.5 When Plasticity is Helpful: Cancer Resensitization and Regeneration Therapies

Cancer cell plasticity is usually a curse, since it leads to metastasis, the development of cancer stem cells, and drug resistance. However, there are a number of cases when the “miraculous weapon” of cancer cells, their plasticity, turns against them. As a first example of this situation, the metastasis‐prone cancer mesenchymal state becomes sensitive to quite a few drugs, such as inhibitors of the IRE‐alpha–MKK4 arm of the endoplasmic reticulum stress pathway, BET inhibitors, or ferroptosis (e.g., using the combination therapy of SCD and GPX) [122, 123, 124]. As a second example, drug resistance can be re‐induced by “drug holidays,” i.e., periods when the drug administration is ceased [146, 147]. As a third example, when cancer cells develop drug resistance towards a specific drug, they often become sensitive to another [3]. Thus, the development of shorter time windows for treatments, through careful monitoring of adequate biomarkers and by changing the therapy to a quite different mode of attack, may provide a rich repertoire of sequential therapies in the future—therapies that re‐sensitize the drug‐resistant cancers.

The increase of cell plasticity is a key goal of regeneration therapies, which have become increasingly used in the treatment of diabetes and neurodegenerative diseases. Beta cell regeneration often uses the redifferentiation of highly plastic pancreatic ductal or beta cell progenitor cells [154, 163]. Another route of beta cell regeneration is the transdifferentiation of other cells (such as alpha cells or stem cells), which can be achieved by “transdifferentiation cocktails,” gradually diminishing the initially high plasticity of cells targeted by the treatment [21]. Very recently, stem cell therapy became rather promising for treating Alzheimer's and Parkinson's disease [24, 176, 177, 178].

### 6.6 System Level and Single Cell Network Data and Models: Promises of the Future

Over the past few years, both protein–protein interaction network (interactome) and signaling network data have expanded to encompass the entire human proteome [183, 184, 185]. In addition, system‐level network dynamic models (covering 16% to 40% of the human proteome) have been applied to investigate therapy resistance, perform in silico clinical trials, and conduct large, in silico drug combination screens [148, 186, 187, 188, 189]. However, with only a few exceptions [47, 50], the studies on network plasticity used networks covering only a small part of the proteome, or dynamic models restricted to only a relatively small number of network nodes. System‐level network models are extremely useful to find novel cancer plasticity‐related signaling pathways and pathway cross‐talks; novel cancer cell “differentiation cocktails”; sequential therapy targets, timing, and biomarkers, as well as novel resensitization therapies. The use of system‐level network data and models in the treatment of diabetes and neurodegeneration is a promising future research trend [148, 190, 191, 192, 193].

Important developments also include time series analysis of single‐cell RNA‐sequencing data, giving multiple snapshot analyses of the underlying gene regulatory network [194]. Spatial and time‐series single‐cell multiomics dynamic network analysis is a rapidly increasing, very promising area of future studies [195, 196].

## 7 Conclusion

An initial increase in cell plasticity accompanies most cellular adaptive processes. This increase is typically followed by a subsequent decrease in plasticity. Cancer‐induced cell reprogramming often involves the high plasticity of epithelial–mesenchymal transition (EMT), which may lead to the formation of extremely plastic cancer stem cells. Cell plasticity changes govern beta cell regeneration therapies in diabetes, as well as the very recently expanding stem cell therapies in Alzheimer's and Parkinson's diseases.

Plasticity changes of protein structure networks show how simple macromolecular structures can already display a “molecular intelligence.” Increased plasticity of intrinsically disordered proteins (IDPs), the integrative plasticity of multiple phosphorylation events, molecular chaperones, and increased intracellular water content all contribute to the development of cellular plasticity. Importantly, 12 out of the 55 cell plasticity‐related drug targets in cancer are IDPs (Table 1). Not surprisingly, IDPs also play a key role in EMT.

The size of protein–protein interaction networks and signaling networks has recently reached the entire human proteome. Plasticity at the cellular level is often induced by intermodular connections (e.g., cross‐talks between signaling pathways). These adaptation‐specific, “noncanonical” network edges often connect distant network regions. Adaptation induces novel connections between network nodes at the network periphery and those in the network core. This may cause a remodeling of the network core, which induces cellular reprogramming.

As a key point, this review reveals how this expanding organized network data structure can be used to develop novel therapeutic options: 1) discovering and blocking cancer plasticity‐related signaling pathways and cross‐talks; 2) using the plasticity of EMT to avoid the highly plastic intermediate EMT states and cancer stem cell‐directed EMT; 3) designing novel differentiation therapies (such as directing EMT towards adipocytes and differentiating cancer stem cells) and 4) eradicating pre‐existent, plastic, drug‐resistant cells by sequential therapies. Table 1 lists 55 plasticity‐related cancer drug targets, where 20 have already approved drugs, 9 have investigational drugs, and 26 are drug target candidates.

There has been a recent expansion of pancreatic beta cell regeneration therapy for type 1 and type 2 diabetes, as well as stem cell therapies in Alzheimer's and Parkinson's diseases. Network analysis offers great assistance in discovering key intermodular connections and master regulators to protect existing beta cells and neurons, as well as in designing novel cell differentiation cocktails to regenerate them. Combinations of beta‐cell endoplasmic reticulum stress or beta‐amyloid, tau protein, and alpha‐synuclein aggregation inhibitors can also be designed using network analysis.

Examination of disease‐specific, novel intermodular connections may lead to highly specific edgetic therapies that target these connections. A more detailed analysis of network core and network periphery changes will offer a novel understanding of disease‐specific plasticity changes and may lead to novel drug candidates. Blocking plasticity‐induced alternative signaling pathways, network‐designed extension of differentiation and sequential therapies, as well as identifying novel sensitive points of drug‐resistant cancer cells (such as ferroptosis), are emerging as new hopes in anti‐cancer therapies. Finally, the use of recently available proteome‐wide network data and models, which offer in silico drug combination screens and in silico clinical trials, will give a new burst of medical research expanding all the therapeutic options listed in this review.

**Note added in Proof**

The rapidly increasing field gave several important discoveries between the acceptance and publication of this manuscript. Cancer, diabetes and neurodegeneration are, in fact network diseases as described by Hans Westerhoff's highly insightful paper [197] showing that plastic molecular networks can be regarded as efficient molecular neuronal networks. Meena et al., [198] showed that five major interconnected axes of phenotypic plasticity in ER‐positive breast cancer: metabolic reprogramming, EMT plasticity, luminal‐basal switching, stemness and drug resistance drive one another. TNF and Oncostatin M can drive proneural to mesenchymal transition of glioblastoma tumor cells.[199] HNF1B was implicated as a transcription factor potentially involved in the plasticity of hepatocellular carcinoma cell heterogeneity and transformation into intrahepatic cholangiocarcinoma.[200] The EMT network was extended by the addition of a redox‐adhesion‐exosome hub linking EMT plasticity to ferroptosis.[201] The NSUN protein family (i.e. NOP2/Sun RNA methyltrasnferases), mediators of 5‐methylcytosine RNA methylation of a large variety of RNAs emerged as central players of tumorigenesis and maintenance of cancer cell plasticity.[202] Both HNF1B and NSUN can be regarded as plasticity related potential drug targets.

## Conflicts of Interest

Peter Csermely and Mark Kerestely report financial support provided by the Ministry for Innovation and Technology in Hungary. Daniel V. Veres reports a relationship with Turbine Ltd. that includes employment and equity or stocks. Other authors declare that they have no known competing financial interests or personal relationships that could have appeared to influence the work reported in this paper.

## Data Availability

The authors have nothing to report.
